# The Commissariat

**Published:** 1902-12-13

**Authors:** 


### THE COMMISSARIAT.

### THE ROAST BEEF OF OLD ENGLAND.

That most of the beef we eat is not English is a fact of which all but those who do not put awkward inquiries to their butchers are aware! If a side of beef is labelled "home fed" we may, for the sake of charity, believe the statement But, for the most part, our beef is American. This has long been a grievance to agriculturists and their well-wishers, but it is to be feared that the well-wishers will no longer have the support of the public. Recently, in the House of Commons, Sir John Leng drew the attention of Mr. Hanbury, the Minister of Agriculture, to a report by the veterinary surgeon of the Corporation of Glasgow, which shows that the home-bred ox is by no means the most desirable as an article of food. A great many foreign cattle are landed at Glasgow, and in accordance with the regulations at present in force, must be slaughtered immediately. In 1901 there were killed at Glasgow 49,881 cattle, imported from Canada and the United States, of which 66 were found to be diseased. In the Glasgow slaughter-houses there were killed during the same year home-bred cattle to the number of 46,784, and the number of diseased animals among these amounted to 6,332. That means that only one out of 4,000 Canadian and States cattle were diseased, as against one out of eight home ones. If the figures of Glasgow hold good for the rest of the kingdom, we cannot but say "Give us American beef." The Glasgow surgeon whose report was quoted is of opinion that the absence of disease in the foreign cattle is due to the greater liberty which they enjoy, being more in the open air, and ranging freely over a wide tract of country. These conditions, Mr. Hanbury feared, it would be impossible to reproduce in their entirety at home. But if that be so, can we expect the position of home-grown cattle, and those who keep them, to improve? If these figures come to be generally known, will not purchasers ask for American beef in preference to English? It may not be so palatable, neither so tender nor so juicy, but, at least, it is safe; it is not diseased. There is some excuse—at least there is a comprehensible plea—for the protection of home industries when it can be shown that the foreign and cheaper article is inferior to the home-made. But when, as in this case, it is superior, all we can do is to advise the native competitor to bring his goods up to the foreign level. Then we will support him gladly.

